# Structure of the JmjC domain-containing protein NO66 complexed with ribosomal protein Rpl8

**DOI:** 10.1107/S1399004715012948

**Published:** 2015-08-28

**Authors:** Chengliang Wang, Qiongdi Zhang, Tianrong Hang, Yue Tao, Xukai Ma, Minhao Wu, Xuan Zhang, Jianye Zang

**Affiliations:** a Hefei National Laboratory for Physical Sciences at Microscale and School of Life Sciences, Collaborative Innovation Center of Chemistry for Life Science, University of Science and Technology of China, 96 Jinzhai Road, Hefei, Anhui 230026, People’s Republic of China; b Key Laboratory of Structural Biology, Chinese Academy of Sciences, Hefei, Anhui 230026, People’s Republic of China; c Institute for Pediatric Translational Medicine, Shanghai Children’s Medical Center, 1678 Dongfang Road, Pudong, Shanghai 200120, People’s Republic of China

**Keywords:** NO66, complex structure, catalytic mechanism, substrate recognition

## Abstract

The JmjC domain-containing proteins belong to a large family of oxygenases possessing distinct substrate specificities which are involved in the regulation of different biological processes, such as gene transcription, RNA processing and translation. Nucleolar protein 66 (NO66) is a JmjC domain-containing protein which has been reported to be a histone demethylase and a ribosome protein 8 (Rpl8) hydroxylase. The present biochemical study confirmed the hydroxylase activity of NO66 and showed that oligomerization is required for NO66 to efficiently catalyze the hydroxylation of Rpl8. The structures of NO66^176–C^ complexed with Rpl8^204–224^ in a tetrameric form and of the mutant protein M2 in a dimeric form were solved. Based on the results of structural and biochemical analyses, the consensus sequence motif NH*X*H recognized by NO66 was confirmed. Several potential substrates of NO66 were found by a *BLAST* search according to the consensus sequence motif. When binding to substrate, the relative positions of each subunit in the NO66 tetramer shift. Oligomerization may facilitate the motion of each subunit in the NO66 tetramer and affect the catalytic activity.

## Abstract 2

The structure of the complex of NO66 and Rpl8 was solved in the native state and NO66 recognizes the consensus motif NH*X*H . Tetramerization is required for efficient substrate binding and catalysis by NO66.

## 1. Introduction   

The nonhaem α-ketoglutarate (αKG)/Fe^2+^-dependent oxy­genases were originally identified as prolyl and lysyl hydroxylases that have essential roles in collagen biosynthesis (Myllyharju & Kivirikko, 2004 ▸; McDonough *et al.*, 2010 ▸). Subsequently, αKG/Fe^2+^-dependent oxygenases were found to be widely distributed in most organisms and were recognized as a superfamily of enzymes catalyzing various oxidation reactions. These enzymes bear similar catalytic domains folded into double-stranded β-helical (DSBH) structures with a conserved His-*X*-Glu/Asp-*X*_*n*_-His motif responsible for the coordination of Fe^2+^ (Que, 2000 ▸; Clifton *et al.*, 2006 ▸). Other motifs or domains surrounding the catalytic core structure further differentiate these enzymes into subfamilies. These domains may be involved in the regulation of enzymatic activity, thus acting to determine the function of the individual enzymes (McDonough *et al.*, 2010 ▸).

The JmjC domain-containing proteins comprise a subclass of αKG/Fe^2+^-dependent oxygenases which possess distinct substrate specificities and participate in various cellular processes, including regulation of gene expression (Chen *et al.*, 2006 ▸; Whetstine *et al.*, 2006 ▸), mRNA splicing (Webby *et al.*, 2009 ▸) and tRNA modification (Kato *et al.*, 2011 ▸). A large subfamily of JmjC domain-containing proteins function as histone demethylases, which participate in many fundamental biological processes such as transcriptional regulation and epigenetic inheritance (Jenuwein & Allis, 2001 ▸; Martin & Zhang, 2005 ▸). Other JmjC domain-containing proteins catalyze the oxidation of nonhistone proteins and nucleic acids, and have been shown to be essential for the regulation of gene expression, RNA processing and the fidelity of translation (Chowdhury *et al.*, 2009 ▸; Webby *et al.*, 2009 ▸; Kato *et al.*, 2011 ▸).

Nucleolar protein 66 (NO66) is a metazoan JmjC domain-containing protein that is highly conserved throughout evolution and was initially identified as a dual-location intranuclear protein (Eilbracht *et al.*, 2004 ▸). In the nucleus, NO66 was reported to interact with the osteoblast-specific transcription factor osterix (Osx) and to regulate the expression of Osx-dependent genes and bone formation (Sinha *et al.*, 2010 ▸). In addition, recent work showed that NO66 is recruited to the stem-cell genes together with the PRC2 (polycomb repressor complexes 2) complex during differentiation, resulting in gene silencing (Brien *et al.*, 2012 ▸). In both cases, NO66 was reported to be a histone demethylase *in vivo*. Evidence from a recent study indicated that NO66 hydroxylates ribosome protein 8 (Rpl8) and controls the biogenesis of the ribosome (Ge *et al.*, 2012 ▸), which is consistent with the observation that NO66 is a constitutive nucleolar component (Eilbracht *et al.*, 2004 ▸). Moreover, previous work demonstrated that NO66 assembles as a tetramer and that the binding site of NO66 to osterix is located on the surface formed by a hinge region (interface I) from neighbouring subunits in the tetramer (Tao *et al.*, 2013 ▸). Although tetramer formation has been shown to be important for the interaction of NO66 with osterix and the regulation of gene transcription, whether tetrameric assembly is required for its catalytic activity remains unknown. Thus, further investigation of the enzymatic activity of NO66 is required. While this manuscript was under preparation, Chowdhury and coworkers published the structure of NO66 in complex with an Rpl8 peptide using a disulfide-based cross-linking strategy, suggesting a potential interaction between NO66 and Rpl8 (Chowdhury *et al.*, 2014 ▸). However, further research is required to understand the mechanism of interaction between NO66 and Rpl8 in the native state.

Here, we performed biochemical assays to confirm that NO66 hydroxylates Rpl8. In our assays, oligomerization is required for NO66 to hydroxylate Rpl8 efficiently. In addition, we determined the structures of NO66^176–C^ complexed with Rpl8^204–224^ and the mutant protein M2 lacking one of the dimerization interfaces in NO66. Based on structural and biochemical analyses, we confirmed that NO66 recognizes a consensus sequence motif and found that the relative positions of each subunit in the NO66 tetramer change upon substrate binding. Based on these observations, we proposed that oligomerization could affect the catalytic activity of NO66.

## 2. Methods   

### 2.1. Protein purification and crystallization   

The C-terminal fragment of wild-type human NO66 (NO66^176–C^) was cloned, expressed and purified as described previously (Zhou *et al.*, 2012 ▸; Tao *et al.*, 2013 ▸). In brief, NO66^176–C^ or its mutants were cloned into pET-28a(+) (Novagen) with a His-SUMO tag fused at the N-terminus and were expressed in *Escherichia coli* Rosetta 2 (DE3) cells. The target proteins were purified using Ni–NTA resin (GE Healthcare). The His-SUMO tag was removed by TEV protease and the proteins were further purified using a HiLoad 10/300 Superdex 200 column (GE Healthcare) in buffer *A* (20 m*M* Tris–HCl pH 7.5, 200 m*M* NaCl). The eluted fractions corresponding to target proteins were collected and concentrated for further use.

Crystals of NO66^176–C^ in complex with Rpl8^204–224^ peptide (human ribosomal protein L8) were obtained using the sitting-drop vapour-diffusion method at 295 K. Before crystallization, Ni^2+^, NOG (*N*-oxalylglycine, an analogue of α-ketoglutarate) and Rpl8 peptide were added to the protein solution with a final molar ratio of 1:4:10:15 (protein:NOG:Ni^2+^:peptide) and the mixture was incubated for about 1 h. The crystal used for data collection was grown in a solution consisting of 0.1 *M* imidazole pH 6.5, 0.5 *M* sodium acetate trihydrate. Crystals of the M2 mutant in complex with Ni^2+^ were grown using the same procedures. The final crystallization condition for M2 was 1.0 *M* (NH_4_)_2_HPO_4_, 0.1 *M* acetate pH 4.5.

### 2.2. Data collection and structure determination   

The crystals were gradually transferred into cryoprotectant solution supplemented with 20%(*v*/*v*) glycerol and flash-cooled in liquid nitrogen. X-ray diffraction data were collected at 100 K on beamline BL17U1 at the Shanghai Synchrotron Radiation Facility. Diffraction data were indexed, integrated and scaled using *iMosflm* (Battye *et al.*, 2011 ▸) and *POINTLESS* and *SCALA* (Evans, 2006 ▸) from the *CCP*4 suite (Winn *et al.*, 2011 ▸). The structure of NO66^176–C^ in complex with Rpl8 peptide was solved by molecular replacement (McCoy *et al.*, 2007 ▸) using the structure of NO66^183–C^ (PDB entry 4e4h; Y. Tao, M. Wu, K. M. Sinha & J. Zang, unpublished work) as a search model. The structure was refined to 2.2 Å resolution using *REFMAC*5 (Murshudov *et al.*, 2011 ▸) and *Coot* (Emsley & Cowtan, 2004 ▸). Similar methods were used to refine the structure of the M2 mutant. The final structural models were validated using *PROCHECK* (Laskowski *et al.*, 1993 ▸). The data-processing and structure-determination statistics are listed in Table 1 ▸. All of the structural figures were prepared using *PyMOL* (http://www.pymol.org).

### 2.3. GST pull-down assay   

Various fragments of human Rpl8 were cloned into the pGEX-6P-1 vector and overexpressed in *E. coli* BL21 (DE3) cells. The cells were lysed in 1 ml buffer *B* (50 m*M* HEPES pH 7.5, 300 m*M* NaCl, 0.1% Triton X-100). The lysates were incubated with 20 µl GST beads pre-equilibrated in buffer *B*. After washing three times, GST-Rpl8 fragments immobilized with GST beads were incubated with purified NO66^176–C^ or mutants at 277 K for 1 h. The beads were washed with buffer *B* four times and boiled with SDS sample buffer. The proteins retained on the glutathione Sepharose beads were analyzed using SDS–PAGE.

### 2.4. Hydroxylation activity analysis   

NO66^176–C^ (10 µ*M*) was incubated with Rpl8^204–224^ peptide (100 µ*M*) in 100 µl buffer *C* [50 m*M* HEPES pH 7.5, 300 m*M* NaCl, 0.5 m*M* TCEP, 200 µ*M* αKG, 100 µ*M* Fe(NH_4_)_2_(SO_4_)_2_, 200 µ*M* ascorbic acid] at room temperature for 60 min (Ge *et al.*, 2012 ▸). The reaction was quenched by the addition of 10 µl 0.1% TFA (trifluoroacetic acid) and the insoluble material was removed by centrifugation. The supernatant was desalted using C_18_ ZipTips (Millipore; Chen *et al.*, 2006 ▸). The bound material was eluted from the C_18_ ZipTips with 70% aceto­nitrile/0.1% TFA with or without α-cyano-4-hydroxycinnamic acid. The eluted samples were used for MALDI-TOF MS or LC-MS/MS analysis.

### 2.5. Size-exclusion chromatographic analysis   

The purified NO66^176–C^ or mutant (M1, M2 and M3) proteins were loaded onto a HiLoad 10/300 Superdex 200 column (GE Healthcare) pre-equilibrated with the column buffer (20 m*M* Tris–HCl pH 7.5, 200 m*M* NaCl) and eluted using the same buffer. The molecular weight of each protein was estimated according to the elution volume.

### 2.6. Isothermal titration calorimetry (ITC)   

ITC measurements were performed at 287 K as reported previously (Ruan *et al.*, 2012 ▸). 40 µl αKG (500 µ*M* stock solution) was injected into a sample cell containing 20 µ*M* wild-type NO66 or mutant protein fused to the SUMO tag in buffer *D* (50 m*M* Tris–HCl pH 7.5, 300 m*M* NaCl, 20 µ*M* NiCl_2_). To measure the affinity of substrate binding, 40 µl GST-Rpl8^193–C^ (270 µ*M* stock solution) was injected into a sample cell containing 15 µ*M* protein in buffer *D* supplemented with 60 µ*M* αKG. The ITC measurements were fitted to a one-site binding model using the *Origin* software (MicroCal Inc.).

## 3. Results   

### 3.1. Oligomerization of NO66 is required for substrate binding and catalytic activity   

**Figure 1:**
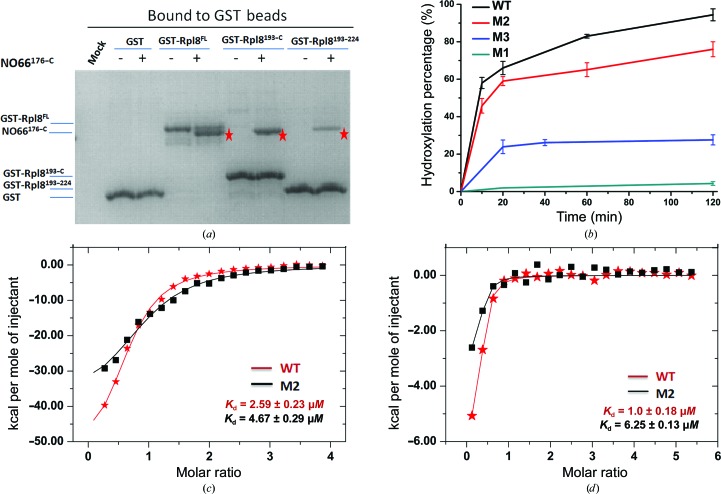
Oligomerization of NO66 is required for substrate binding and catalysis. (*a*) GST pull-down of NO66^176–C^ by different fragments of Rpl8 fused to GST. Asterisks indicate the band corresponding to NO66^176–C^. (*b*) A comparison of the efficiency of wild-type NO66 and mutants. The products of the hydroxylation reaction were detected by LC-MS/MS and the data are semi-quantitative. (*c, d*) Comparison of the binding of wild-type NO66^176–C^ (red) and mutant protein M2 (black) to GST-Rpl8^193–C^ (*c*) and αKG (*d*). The ITC method was used to analyze the binding affinities.

NO66 was recently identified as a protein hydroxylase that is capable of catalyzing histidyl hydroxylation of Rpl8 (Ge *et al.*, 2012 ▸). To confirm the activity of NO66, we evaluated the interaction between NO66 and Rpl8. We performed *in vitro* pull-down assays with several fragments of Rpl8 fused to GST. As expected, the C-terminal fragment of Rpl8 binds to NO66^176–C^ (Fig. 1 ▸ *a*). In addition, a short region of Rpl8 spanning amino-acid residues 193–224 is sufficient to interact with NO66^176–C^ (Fig. 1 ▸ *a*). Next, we examined the histidyl hydroxylation activity of NO66^176–C^ by using a synthetic peptide containing 21 amino-acid residues of Rpl8 (204–224; referred to as Rpl8^204–224^) as the substrate. After incubation with NO66^176–C^, the molecular weight of Rpl8^204–224^ had a +16 Da shift, indicating the possible occurrence of hydroxylation (Supplementary Fig. S1*a*). Further analyses showed that His216 of Rpl8 is hydroxylated by NO66^176–C^ (Supplementary Fig. S1*b*). Our activity assays confirmed that NO66 is a protein histidyl hydroxylase.

Our previous analyses demonstrated that NO66^176–C^ exists as a tetramer in solution (Tao *et al.*, 2013 ▸). Oligomerization is necessary for NO66 to associate with Osx and this represses the expression of Osx-dependent genes (Tao *et al.*, 2013 ▸). However, whether tetramer formation is also necessary for the catalytic activity of NO66 remains unknown. Therefore, we generated several mutants to disrupt the oligomerization of NO66 and compared their catalytic activities with that of the wild-type protein. As reported previously, we had already produced two mutant proteins, M1 and M2, lacking dimer interfaces I and II (Supplementary Fig. S2), and they exist as a monomer and a dimer, respectively (Tao *et al.*, 2013 ▸). Because the tetramer of NO66 is formed through dimer interfaces I and II, disruption of these two dimer interfaces would lead to two types of dimers (Supplementary Fig. S2). Unexpectedly, we only obtained monomeric NO66 on the deletion of dimer interface I. Therefore, we produced a third mutant, M3, by mutating three amino-acid residues (F450A/R452A/P455A) in dimer interface I, and this mutant forms a dimer in solution (Supplementary Fig. S2).

After purification of the NO66 mutants, we measured their hydroxylation activity *in vitro*. The M1 mutant lost catalytic activity. Compared with wild-type NO66, the activity of the M2 protein decreased and the activity of the M3 protein decreased even further (Fig. 1 ▸ *b*). These results indicate that oligomerization is necessary for catalytic activity of NO66, and that the dimer interface I of NO66 plays a more important role in catalysis than dimer interface II. In addition, we used ITC assays to measure the binding of wild-type and mutant NO66 to its substrate GST-Rpl8^193–C^ and cofactor αKG. The binding of wild-type NO66 to GST-Rpl8^193–C^ is the strongest (*K*_d_ = 2.59 ± 0.23 µ*M*). The M2 protein binds to GST-Rpl8^193–C^, but with a nearly twofold weaker affinity (*K*_d_ = 4.67 ± 0.29 µ*M*). The interaction between GST-Rpl8^193–C^ and the M1 or M3 mutants may be very weak and may not be detected by ITC assays (Fig. 1 ▸ *c* and Supplemetary Fig. S3*a*). We also investigated the binding of these proteins to the cofactor αKG. Similarly, the binding affinity of wild-type NO66 to αKG is the highest (*K*_d_ = 1.0 ± 0.18 µ*M*). The M2 mutant binds to αKG with an approximately sixfold lower affinity (*K*_d_ = 6.25 ± 0.13 µ*M*). The binding of the M1 and M3 mutants to αKG is almost undetectable under these conditions (Fig. 1 ▸ *d* and Supplementary Fig. S3*b*). Collectively, these data suggested that the oligomerization of NO66 is required for substrate and cofactor binding, which may further affect the reaction catalyzed by NO66.

### 3.2. Overall structure of NO66^176–C^ complexed with Rpl8 peptide   

To better understand the catalytic mechanism of NO66, we determined the structure of NO66 in complex with a synthetic peptide derived from Rpl8 (residues 204–224, referred to as Rpl8^204–224^). The fragment of NO66 that we used for structural analysis lacks the N-terminal 175 amino acids (referred to as NO66^176–C^). The Fe^2+^ ion and αKG are naturally accommodated in the active site of NO66. To determine the complex structure of NO66^176–C^ and Rpl8^204–224^, Fe^2+^ ion and αKG were substituted by Ni^2+^ and NOG to prevent the reaction catalyzed by NO66 during crystallization. The statistics of data collection and refinement are shown in Table 1 ▸.

**Figure 2:**
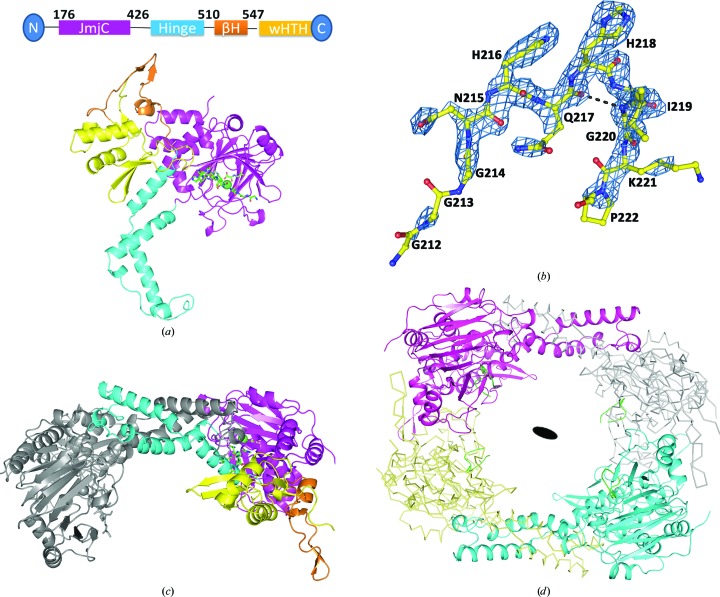
Overall structure of NO66^176–C^ complexed with Rpl8 peptide. (*a*) Cartoon representation of the crystal structure of NO66^176–C^ complexed with Rpl8^204–224^, a peptide derived from Rpl8. The JmjC domain, the dimerization hinge domain, the β-hairpin motif and the C-terminal wHTH domain are shown in pink, cyan, orange and magenta, respectively. The Rpl8 peptide is coloured green. (*b*) The bound peptide Rpl8^204–224^ is shown as sticks. The *F*_o_ − *F*_c_ OMIT electron-density map for bound substrate was contoured at 3.0σ. A cartoon representation of the dimeric (*c*) and tetrameric (*d*) structure of NO66^176–C^ in complex with Rpl8^204–224^ is shown. A dimer is present in the asymmetric unit. Two dimers related by crystallographic twofold symmetry pack against each other to form the tetramer. The filled oval indicates the twofold symmetry axis.

Similar to previous reports (Tao *et al.*, 2013 ▸), the structure of NO66^176–C^ consists of four regions: the JmjC domain (residues 176–426), the hinge-domain region (residues 427–510), a β-hairpin motif (residues 511–547) and the C-terminal wHTH motif (residues 548–641) (Fig. 2 ▸ *a*). In the catalytic centre of NO66^176–C^, electron density corresponding to Rpl8 residues 212–222 is clearly visible (Figs. 2 ▸ *a* and 2 ▸ *b*). There are two molecules in one asymmetric unit that form a dimer through dimer interface I (hinge domain; Fig. 2 ▸ *c*). These two molecules are similar to each other, with a main-chain root-mean-square deviation (r.m.s.d.) of 0.22 Å. Two neighbouring dimers related by the crystallographic twofold rotation symmetry pack against each other to form the tetramer (Fig. 2 ▸ *d*).

### 3.3. Structural basis for specific binding of Rpl8 by NO66   

**Figure 3:**
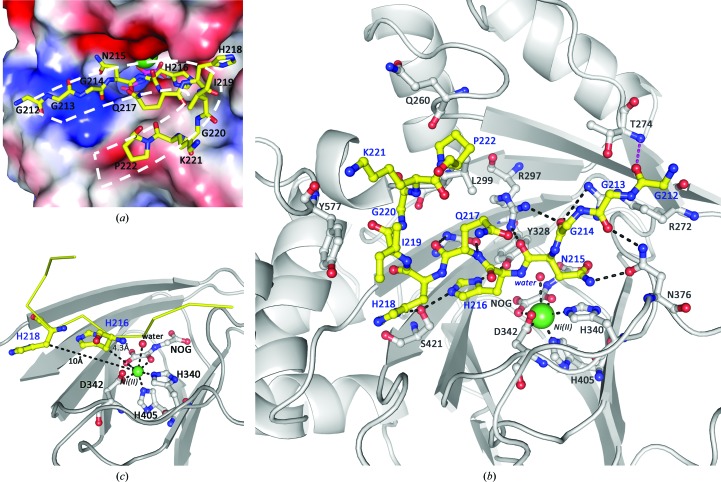
Specific recognition of Rpl8 by NO66. (*a*) A surface representation of the active site of NO66 is shown. Positively charged, negatively charged and neutral areas are shown in blue, red and white, respectively. The Rpl8^204–224^ peptide adopts a U-shaped conformation in the active site of NO66. (*b*) A detailed view of the interaction network of NO66 with Rpl8^204–224^ and NOG. Rpl8^204–224^ and the amino-acid residues of NO66 interacting with Rpl8^204–224^ are shown as stick models. Ni^2+^ and water molecules are shown as spheres. NO66, Rpl8^204–224^, NOG, Ni^2+^ and water molecules are coloured grey, yellow, green, magenta and red, respectively. (*c*) His216 is the hydroxylation site in Rpl8 catalyzed by NO66. The distances between the C^β^ atoms of His216 and His218 and the Ni^2+^ ion in the active site are shown.

In the complex structure of NO66^176–C^ and Rpl8^204–224^ only residues 212–222 of Rpl8 are visible in the electron-density map. The other regions of this peptide are invisible, possibly because of high flexibility. From the complex structure, it is apparent that the Rpl8^204–224^ peptide bound to NO66^176–C^ in a cleft located in the centre of the JmjC domain (Fig. 3 ▸ *a*). The overall surface area of NO66 covered by the bound peptide is about 659 Å^2^. The Rpl8^204–224^ peptide adopts a U-shaped conformation and penetrates deeply into a cleft (Fig. 3 ▸ *a*). Residue Gln217 points to the outside of the binding pocket, leading to a bulge shape formed in the middle of the U-shaped Rpl8^204–224^ peptide. This bulge-shaped structure is stabilized by a hydrogen bond formed between the backbone carbonyl group of Gly217 and the backbone amide group of Gly220 of Rpl8^204–224^ (Fig. 2 ▸ *b*).

**Figure 4:**
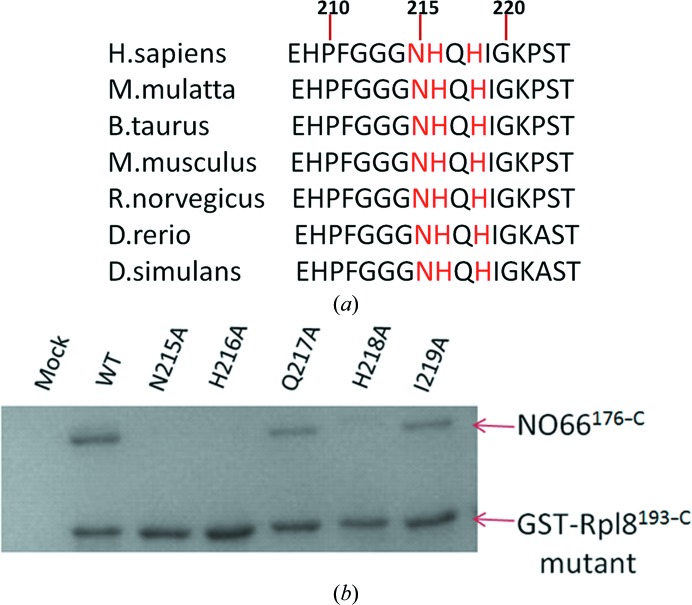
NO66 recognizes Rpl8 through a consensus motif. (*a*) A sequence alignment of the fragment in Rpl8 which is recognized by NO66 is shown. The accession numbers of the protein sequences are *Homo sapiens*, NP_150644 (human); *Macaca mulatta*, NP_001253030 (rhesus macaque); *Bos taurus*, NP_001029797 (cattle); *Mus musculus*, NP_036183 (mouse); *Rattus norvegicus*, NP_001030088 (rat); *Danio rerio*, NP_957007 (fish); *Drosophila simulans*, NP_728756 (fly). The residues that are critical for the binding of Rpl8 to NO66 are highlighted in red. (*b*) Pull-down of NO66^176–C^ by wild-type Rpl8^193–C^ and mutants of Rpl8^193–C^ fused to GST are shown.

By analysis of the complex structure of NO66^176–C^ and Rpl8^204–224^, we found that the Rpl8^204–224^ peptide is tightly embedded in the binding cleft, especially near the four residues (Asn215, His216, Gln217 and His218) located in the middle of the peptide. The N-terminal three glycine residues are on the surface of the entrance to the binding cleft. The backbone carbonyl groups of these glycine residues form hydrogen bonds to the side chains of Arg272, Thr274 and Asn376 of NO66 (Fig. 3 ▸ *b*). Residue Asn215 of the Rpl8^204–224^ peptide forms hydrogen bonds to Arg297 and Asn376 of NO66 *via* the backbone carbonyl group and side chain. Further hydrogen bonds are observed between Gln217 of the Rpl8^204–224^ peptide and Asn326 of NO66. The four C-terminal residues Ile219, Gly220, Lys221 and Pro222 of the Rpl8^204–224^ peptide make van der Waals interactions with the side chains of Gln260, Leu299 and Tyr577 of NO66. In the active site of NO66, two histidine residues of the Rpl8^204–224^ peptide, His216 and His218, are accommodated in two deeply buried pockets. His218 forms a hydrogen bond to Ser421 and the distance between the C^β^ atom and the Ni^2+^ ion is 10 Å. His216 is directed to the active centre by forming hydrogen bonds to Tyr328 and Ser421. The C^β^ atom of His216 is only 4.3 Å away from the Ni^2+^ ion in the active site (Fig. 3 ▸ *c*). The distance is close enough for Fe^2+^ in native NO66 to activate the coordinated O atom and catalyze the hydroxylation reaction. This observation is consistent with the results of biochemical analyses showing that NO66 hydroxylates the C^β^ atom of His216 (Ge *et al.*, 2012 ▸). Alignment of the amino-acid residues involved in the interactions between NO66 and Rpl8 demonstrates that these residues are highly conserved in both proteins from different species, implying the importance of these interactions in anchoring Rpl8 to the active site of NO66 and in presenting the C^β^ atom of His216 in Rpl8 for hydroxylation (Fig. 4 ▸ *a*, Supplementary Fig. S4).

### 3.4. NO66 recognizes Rpl8 through a conserved motif   

Analysis of the complex structure of NO66^176–C^ and the Rpl8^204–224^ peptide demonstrated that NO66 binds to Rpl8 *via* hydrogen bonds and van der Waals interactions. The side chains of Asn215, His216 and His218 form hydrogen bonds to amino-acid residues from NO66, which determines the specificity of the recognition between NO66 and Rpl8. The N-terminal three glycine residues are highly flexible and provide an opportunity for Rpl8 to fit into the shallow groove at the entrance of the active site of NO66. Amino-acid sequence alignment shows that the region of Rpl8 recognized by NO66 is highly conserved (Fig. 4 ▸ *a*). Previous biochemical studies reported that the NH*X*H motif is preferred for NO66 hydroxylation (Ge *et al.*, 2012 ▸). Based on our structural analysis and the result of multiple sequence alignment (Fig. 3 ▸ and Supplementary Fig. S4), we confirmed that NO66 recognizes the NH*X*H motif (where *X* is any amino-acid residue) and the flexibility of three consecutive glycine residues helps Rpl8 enter the active site of NO66. For further investigation, we performed a series of mutation experiments in Rpl8 and examined their interactions with NO66 by pull-down assays. Compared with wild-type Rpl8, mutating Asn215, His216 and His218 to alanine completely abolished the binding to NO66. In contrast, mutating Gln217 and Ile219 to alanine has only a slight effect on the interaction between the mutant proteins and NO66 (Fig. 4 ▸ *b*). The pull-down assays results are consistent with our structural analysis.

### 3.5. Conformational changes of NO66 induced by substrate binding   

**Figure 5:**
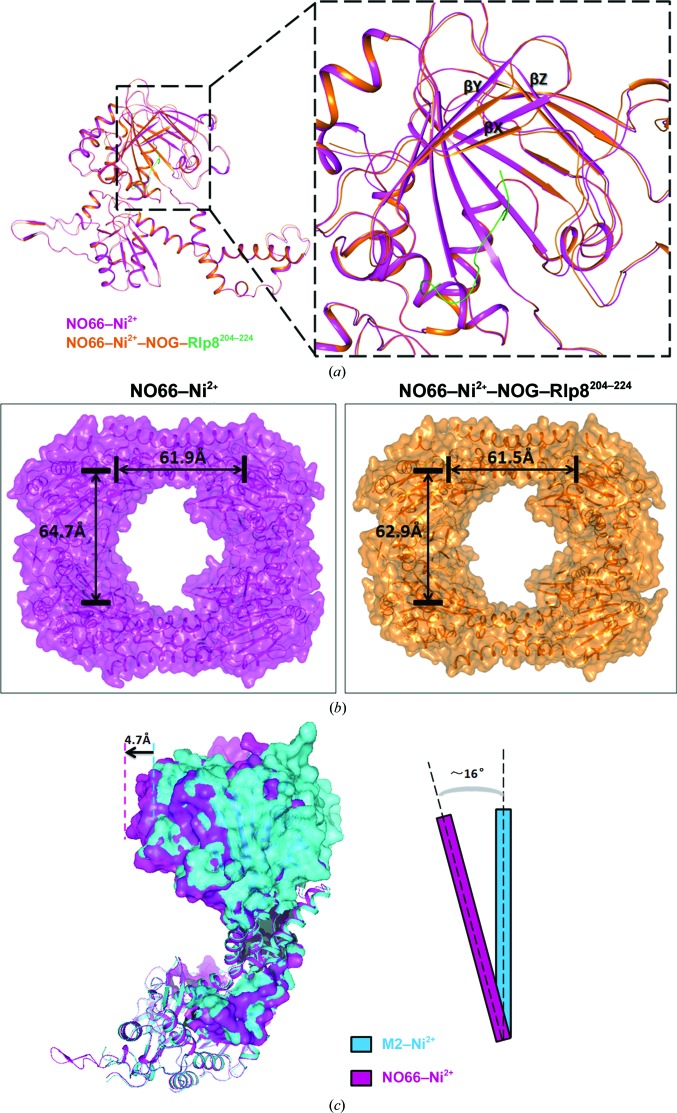
Conformational changes of NO66 are induced by substrate binding. (*a*) A comparison of the complex structure of NO66^176–C^–Rpl8^204–224^ (orange) with NO66^176–C^–Ni^2+^ (magenta) is shown. The enlarged view shows the conformational changes in the active site. The three β-sheets βX (271–274), βY (262–268) and βZ (404–408) and several loops are rearranged upon substrate binding. (*b*) Surface representation of NO66^176–C^–Ni^2+^ (left) and the complex between NO66^176–C^ and Rpl8^204–224^ (right) are shown. The distances between the active sites of NO66 in the two types of dimers are indicated. (*c*) A comparison of the structure of M2 (cyan) with one dimer of wild-type NO66 associated by interface I (magenta) is shown.

Biochemical analysis showed that tetramerization is required for NO66 to catalyze hydroxylation efficiently. To better understand the mechanism, we compared the structure of the complex of NO66^176–C^ and Rpl8^204–224^ with the structure of NO66^176–C^–Ni^2+^ (PDB entry 4e4h). The overall structure of monomeric NO66 is very similar in both NO66^176–C^ complexed with Rpl8^204–224^ and NO66^176–C^–Ni^2+^, with an r.m.s.d. of 0.28 Å for all aligned amino-acid residues (Fig. 5 ▸ *a*). Major conformational changes were observed at the active site. Upon substrate binding, βX (residues 271–274), βY (residues 262–268), βZ (residues 404–408) and several loops shift towards the centre of the substrate-binding site owing to the inter­actions between NO66 and Rpl8 described previously (Fig. 3 ▸ *b*). We further compared the tetrameric structure of the NO66^176–C^–Ni^2+^ and NO66^176–C^–Rpl8^204–224^ complexes. In contrast to the similarity of the monomeric structure, the relative positions of the active sites of each subunit change in the two structures. The distance between the active sites in the dimer linked by interface II is 1.8 Å closer in the complex structure than in NO66^176–C^–Ni^2+^. In contrast, the same distance in the dimer connected by interface I is almost unaltered and the observed difference is only 0.4 Å (Fig. 5 ▸ *b*).

Our biochemical assay results showed that the deletion of interface II resulted in a decrease in the catalytic activity of NO66 (Fig. 1 ▸ *b*). To further understand the role of interface II in the regulation of the activity of NO66, we determined the structure of the mutant protein M2 lacking the interface. As predicted, the M2 protein forms a dimer with interface I. The overall structure of monomeric M2 protein is almost identical to wild-type NO66, with an r.m.s.d. of 0.37 Å (Supplementary Fig. S5). Interestingly, when the dimer structure of the M2 protein was superimposed onto the dimer of NO66 coupled in the same way, we found that one of the M2 molecules in the dimer moves outwards compared with the wild-type protein. When one of the subunits of the M2 dimer was superposed onto NO66, the other subunit of the M2 dimer rotates about 16° and shifts 4.7 Å (Fig. 5 ▸ *c*).

## 4. Discussion   

NO66 was reported to be a dual-location protein found in both the nucleus and the nucleolus (Eilbracht *et al.*, 2004 ▸). Previous studies showed that NO66 is a histone demethylase *in vivo* which is involved in gene-transcription regulation in the nucleus (Sinha *et al.*, 2010 ▸; Brien *et al.*, 2012 ▸; Sinha *et al.*, 2014 ▸). However, recent studies demonstrated that NO66 has protein-hydroxylation activity and could control the biogenesis of ribosomes in the nucleolus (Ge *et al.*, 2012 ▸). Because the function of NO66 remains unclear, we examined its activity in both histone demethylation and protein hydroxylation. We did not observe the demethylation of histone peptides under the conditions usually used for such reactions *in vitro*. By contrast, hydroxylation activity of NO66 was clearly observed in our assays when a synthetic peptide derived from Rpl8 was used as a substrate (Supplementary Figs. S1*a* and S1*b*), which is consistent with previous findings (Ge *et al.*, 2012 ▸; Chowdhury *et al.*, 2014 ▸). However, the possibility could not be excluded that NO66 functions as a demethylase *in vivo* when assisted by other factors or under different conditions.

To gain insight into the molecular mechanisms for the enzymatic activity of NO66, we solved the crystal structure of NO66^176–C^ in complex with Rpl8^204–224^ at 2.2 Å resolution. In the complex structure, Rpl8^204–224^ forms a U-shaped conformation with a bulge-shaped structure in the middle of the peptide. The N- and C-terminus of Rpl8^204–224^ are pushed close to each other and the middle of the peptide protrudes from the end (Fig. 3 ▸ *a*). As a result, His216 and His218 locate in the middle of the Rpl8^204–224^ peptide inserted into the binding pockets and His216 is directed to the active site for hydroxylation. As demonstrated by the complex structure, only the side chains of Asn215, His216 and His218 of Rpl8 form hydrogen bonds to NO66. Point mutations of this motif disrupt the inter­actions between NO66 and Rpl8 (Fig. 4 ▸ *b*), which is consistent with the alanine-scanning results (Ge *et al.*, 2012 ▸).

**Figure 6:**
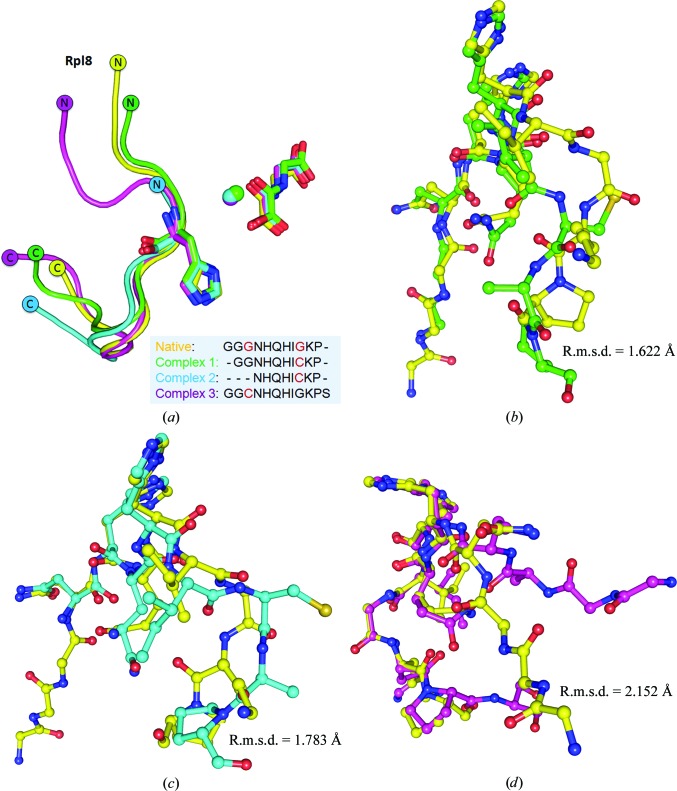
Comparison of structures of the Rpl8 peptide bound to NO66. (*a*) Chowdhury *et al.* (2014 ▸) used a cross-linking strategy to determine the complex structures of NO66 and Rpl8 peptides. In contrast, we determined the structure of NO66^176–C^ in complex with Rpl8^204–224^ in the native state. Cross-linking by disulfide bonds affects the conformation of the Rpl8 peptide bound to NO66. The Rpl8 peptide binds to NO66 in the native state and is coloured yellow. Rpl8 peptides cross-linked to NO66 are coloured green (complex 1; PDB entry 4ccm), blue (complex 2; PDB entry 4ccn) and magenta (complex 3; PDB entry 4cco), respectively. Sequence-alignment results of Rpl8 peptides from these complex structures are shown at the bottom of the panel. Amino-acid residues mutated to cysteine are highlighted in red. (*b*, *c*, *d*) Detailed comparison of the Rpl8 conformation between the native state and three cross-linked structures.

Our native substrate-bound structure of NO66 reveals a different conformation of the Rpl8 peptide compared with the Rpl8 peptides in the previously published cross-linked structures (Chowdhury *et al.*, 2014 ▸), demonstrating the native interaction mechanism between NO66 and Rpl8 (Fig. 3 ▸). Comparison of Rpl8 peptides between our natural state structure and the cross-linked structures (Chowdhury *et al.*, 2014 ▸) shows all-atom r.m.s.d.s of 1.783 Å for complex 1 (PDB entry 4ccm), 1.622 Å for complex 2 (PDB entry 4ccm) and 2.152 Å for complex 3 (PDB entry 4cco) (Fig. 6 ▸). The cross-linked structures show a much higher value of the average *B* factor for the peptides for both the whole chain and the critical residues (Supplementary Table S1), indicating that cross-linked peptides do not stably interact with NO66.

The results obtained from our structural and biochemical analyses are in agreement with recently reported functional studies of NO66 in ribosome biogenesis (Eilbracht *et al.*, 2004 ▸). However, NO66 has also been demonstrated to associate with chromatin and to regulate gene transcription (Sinha *et al.*, 2010 ▸, 2014 ▸). The hydroxylation of Rpl8 is unable to accomplish this task, which suggested that NO66 may have additional substrates. A *BLAST* search identified approximately 20 proteins that contain sequences similar to the consensus motif recognized by NO66 (Supplementary Table S2). These proteins are potential substrates of NO66 and some of them are involved in the regulation of gene transcription. Consequently, we hypothesize that NO66 may have additional substrates *in vivo* and this could affect gene transcription indirectly.

From the results of our biochemical assays, we observed that NO66, when assembled in a tetrameric form, catalyzes the hydroxylation of Rpl8 most efficiently. Disruption of the tetrameric assembly in various ways leads to loss of catalytic activity (Fig. 1 ▸ *b*). Oligomerization has been demonstrated to be an efficient way to control the activity of JmjC domain-containing enzymes. For example, destabilization of the FIH dimer results in an enzymatically in­active monomer (Lancaster *et al.*, 2004 ▸). In addition, disruption of dimerization in Mina53 and YcfD causes a loss of activity (Chowdhury *et al.*, 2014 ▸). These biochemical results reveal that some JmjC domain-containing enzymes exist in oligomeric forms, which might be important for control of their activity.

To further understand the mechanism, we determined the structure of the mutant protein M2 and compared the NO66^176–C^–Rpl8^204–224^ complex structure with the NO66–Ni^2+^ structure (PDB entry 4e4h) and the M2 structure. We found that in the tetrameric structure of NO66^176–C^ complexed with Rpl8^204–224^ the active sites of NO66 in the dimer related by both interface I and interface II move closer to each other upon substrate binding (Fig. 5 ▸ *b*). In addition, Mina53 and YcfD form dimers mediated by interfaces similar to interface I of NO66 (Chowdhury *et al.*, 2014 ▸). Interestingly, we noticed that the distances of the active sites in the dimer of Mina53 (PDB entries 4bu2 and 4bxf) and YcfD (PDB entries 4csw and 4cug) were shortened in the same way when substrates were bound (Supplementary Fig. S6). In addition, the relative positions of the two subunits in the dimer of the mutant protein M2 shift outwards compared with wild-type NO66 (Fig. 5 ▸ *c*). Interface II, which was deleted in M2, pulls these two subunits towards the other two subunits to form an NO66 tetramer. This tetramerization may further facilitate the shifting of the subunits when NO66 binds to its substrate. Although we were unable to determine how such conformational changes affected the activity of these enzymes, disruption of the oligomerization forms leads to a loss of catalytic activity (Fig. 1 ▸ *b*). In this context, we proposed that oligomerization of NO66 might affect the motion of each subunit in the catalytic process and thus control the activity.

## Supplementary Material

PDB reference: NO66^176–C^–Ni^2+^–NOG, 4y33

PDB reference: NO66^176–C^–Ni^2+^–NOG–RPL8^204–224^, 4y3o

PDB reference: NO66, M2 mutant, 4y4r

Supporting Information.. DOI: 10.1107/S1399004715012948/qh5028sup1.pdf

## Figures and Tables

**Table 1:** Data-collection and refinement statistics Values in parentheses are for the highest resolution shell.

	NO66^176C^Ni^2+^NOGRpl8^204224^	M2
Data collection		
Space group	*C*2	*P*2_1_2_1_2_1_
Unit-cell parameters		
*a* ()	88.50	86.06
*b* ()	202.93	144.21
*c* ()	85.68	144.21
= ()	90	90
()	118.94	90
Resolution ()	50.02.2 (2.322.20)	50.03.3 (3.483.30)
No. of unique reflections	62029	27284
Wilson plot *B* factor (^2^)	20.0	73.7
*R*_meas_ † (%)	14.6 (59.7)	11.0 (67.6)
Mean *I*/(*I*)	7.6 (2.4)	12.4 (3.0)
Completeness (%)	98.0 (97.8)	98.8 (98.0)
Multiplicity	3.4 (3.4)	6.2 (5.6)
Refinement		
Resolution ()	50.02.2	50.03.3
*R*_work_ ‡/*R*_free_ § (%)	20.43/26.03	21.81/27.21
No. of atoms		
Protein	7535	7076
Water	561	4
Other ligand	22	6
R.m.s. deviations		
Bond lengths ()	0.014	0.0142
Bond angles ()	1.559	1.641
*B* factors (^2^)		
Protein	24.47	100.35
Water	28.56	61.25
Other ligand	30.67	57.67
Ramachandran plot		
Most favoured regions (%)	96.29	92.73
Additionally allowed regions (%)	3.71	7.27
Outliers (%)	0	0

† *R*_meas_ was estimated by multiplying the conventional *R*_merge_ value by the factor [*N*/(*N* 1)]^1/2^, where *N* is the data multiplicity; *R*_merge_ = 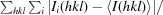 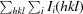, where *I_i_*(*hkl*) is the intensity of the *i*th measurement and *I*(*hkl*) is the mean intensity for that reflection.

‡ *R*_work_ = 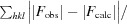 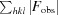, where |*F*_obs_| and |*F*_calc_| are the observed and calculated structure-factor amplitudes, respectively.

§ *R*_free_ was calculated as for *R*_work_ but using the 5.0% of the reflections in the test set.

## References

[bb2] Battye, T. G. G., Kontogiannis, L., Johnson, O., Powell, H. R. & Leslie, A. G. W. (2011). *Acta Cryst.* D**67**, 271–281. 10.1107/S0907444910048675 PMC3069742 21460445

[bb3] Brien, G. L. *et al.* (2012). *Nature Struct. Mol. Biol.* **19**, 1273–1281. 10.1038/nsmb.2449 23160351

[bb4] Chen, Z., Zang, J., Whetstine, J., Hong, X., Davrazou, F., Kutateladze, T. G., Simpson, M., Mao, Q., Pan, C.-H., Dai, S., Hagman, J., Hansen, K., Shi, Y. & Zhang, G. (2006). *Cell*, **125**, 691–702. 10.1016/j.cell.2006.04.024 16677698

[bb5] Chowdhury, R., McDonough, M. A., Mecinović, J., Loenarz, C., Flashman, E., Hewitson, K. S., Domene, C. & Schofield, C. J. (2009). *Structure*, **17**, 981–989. 10.1016/j.str.2009.06.002 19604478

[bb6] Chowdhury, R. *et al.* (2014). *Nature (London)*, **510**, 422–426.

[bb7] Clifton, I. J., McDonough, M. A., Ehrismann, D., Kershaw, N. J., Granatino, N. & Schofield, C. J. (2006). *J. Inorg. Biochem.* **100**, 644–669. 10.1016/j.jinorgbio.2006.01.024 16513174

[bb8] Eilbracht, J., Reichenzeller, M., Hergt, M., Schnölzer, M., Heid, H., Stöhr, M., Franke, W. W. & Schmidt-Zachmann, M. S. (2004). *Mol. Biol. Cell* **15**, 1816–1832. 10.1091/mbc.E03-08-0623 PMC379278 14742713

[bb9] Emsley, P. & Cowtan, K. (2004). *Acta Cryst.* D**60**, 2126–2132. 10.1107/S0907444904019158 15572765

[bb10] Evans, P. (2006). *Acta Cryst.* D**62**, 72–82. 10.1107/S0907444905036693 16369096

[bb11] Ge, W. *et al.* (2012). *Nature Chem. Biol.* **8**, 960–962. 10.1038/nchembio.1093 PMC4972389 23103944

[bb12] Jenuwein, T. & Allis, C. D. (2001). *Science*, **293**, 1074–1080. 10.1126/science.1063127 11498575

[bb13] Kato, M., Araiso, Y., Noma, A., Nagao, A., Suzuki, T., Ishitani, R. & Nureki, O. (2011). *Nucleic Acids Res.* **39**, 1576–1585. 10.1093/nar/gkq919 PMC3045595 20972222

[bb14] Lancaster, D. E., McNeill, L. A., McDonough, M. A., Aplin, R. T., Hewitson, K. S., Pugh, C. W., Ratcliffe, P. J. & Schofield, C. J. (2004). *Biochem. J.* **383**, 429–437. 10.1042/BJ20040735 PMC1133735 15239670

[bb15] Laskowski, R. A., MacArthur, M. W., Moss, D. S. & Thornton, J. M. (1993). *J. Appl. Cryst.* **26**, 283–291.

[bb16] Martin, C. & Zhang, Y. (2005). *Nature Rev. Mol. Cell Biol.* **6**, 838–849. 10.1038/nrm1761 16261189

[bb17] McCoy, A. J., Grosse-Kunstleve, R. W., Adams, P. D., Winn, M. D., Storoni, L. C. & Read, R. J. (2007). *J. Appl. Cryst.* **40**, 658–674. 10.1107/S0021889807021206 PMC2483472 19461840

[bb18] McDonough, M. A., Loenarz, C., Chowdhury, R., Clifton, I. J. & Schofield, C. J. (2010). *Curr. Opin. Struct. Biol.* **20**, 659–672. 10.1016/j.sbi.2010.08.006 20888218

[bb19] Murshudov, G. N., Skubák, P., Lebedev, A. A., Pannu, N. S., Steiner, R. A., Nicholls, R. A., Winn, M. D., Long, F. & Vagin, A. A. (2011). *Acta Cryst.* D**67**, 355–367. 10.1107/S0907444911001314 PMC3069751 21460454

[bb20] Myllyharju, J. & Kivirikko, K. I. (2004). *Trends Genet.* **20**, 33–43. 10.1016/j.tig.2003.11.004 14698617

[bb21] Que, L. Jr (2000). *Nature Struct. Biol.* **7**, 182–184. 10.1038/73270 10700270

[bb22] Ruan, J., Ouyang, H., Amaya, M. F., Ravichandran, M., Loppnau, P., Min, J. & Zang, J. (2012). *PLoS One*, **7**, e35376. 10.1371/journal.pone.0035376 PMC3325965 22514736

[bb23] Sinha, K. M., Yasuda, H., Coombes, M. M., Dent, S. Y. R. & de Crombrugghe, B. (2010). *EMBO J.* **29**, 68–79. 10.1038/emboj.2009.332 PMC2780536 19927124

[bb24] Sinha, K. M., Yasuda, H., Zhou, X. & deCrombrugghe, B. (2014). *J. Bone Miner. Res.* **29**, 855–865. 10.1002/jbmr.2103 PMC3961497 24115157

[bb25] Tao, Y., Wu, M. H., Zhou, X., Yin, W., Hu, B., de Crombrugghe, B., Sinha, K. M. & Zang, J. (2013). *J. Biol. Chem.* **288**, 16430–16437. 10.1074/jbc.M112.446849 PMC3675579 23620590

[bb26] Webby, C. J. *et al.* (2009). *Science*, **325**, 90–93.

[bb27] Whetstine, J. R., Nottke, A., Lan, F., Huarte, M., Smolikov, S., Chen, Z., Spooner, E., Li, E., Zhang, G., Colaiacovo, M. & Shi, Y. (2006). *Cell*, **125**, 467–481. 10.1016/j.cell.2006.03.028 16603238

[bb1] Winn, M. D. *et al.* (2011). *Acta Cryst.* D**67**, 235–242.

[bb28] Zhou, X., Tao, Y., Wu, M., Zhang, D. & Zang, J. (2012). *Acta Cryst.* F**68**, 764–766. 10.1107/S174430911201740X PMC3388916 22750859

