# Supplementary material for: Structure of the JmjC domain-containing protein NO66 complexed with ribosomal protein Rpl8
Source: Acta Crystallogr D Biol Crystallogr. 2015 Aug 28;71(Pt 9):1955–64. doi: 10.1107/S1399004715012948 (PMC4556315; doi:10.1107/S1399004715012948)
Supplement: Supplementary file 1 [file d-71-01955-sup1.pdf]

BIOLOGICAL
CRYSTALLOGRAPHY

Volume 71 (2015)

Supporting information for article:

Structure of JmjC domain-containing protein NO66 complexed with Rpl8

Chengliang Wang, Qiongdi Zhang, Tianrong Hang, Yue Tao, Xukai Ma, Minhao Wu, Xuan Zhang and Jianye Zang

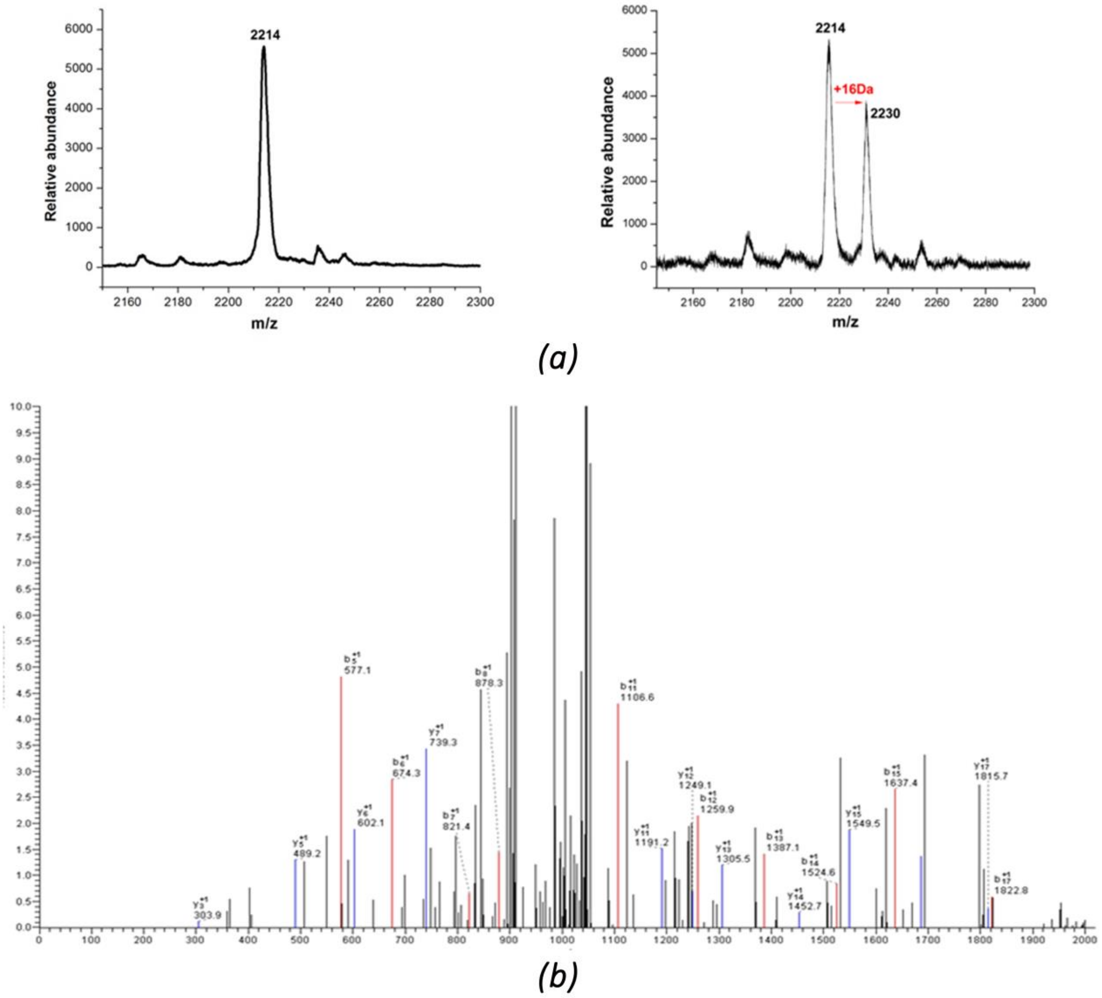

Figure S1 NO66 hydroxylates ribosomal protein L8. (*a*) Mass analysis of *in vitro* hydroxylase activity of NO66. Reaction using Rpl8 peptide combined with NO66 showed a +16Da shift detected by MALDI-TOF analysis. (*b*) LC/MS analysis of rpl8 peptide incubated with NO66 assigned hydroxylation at H216.

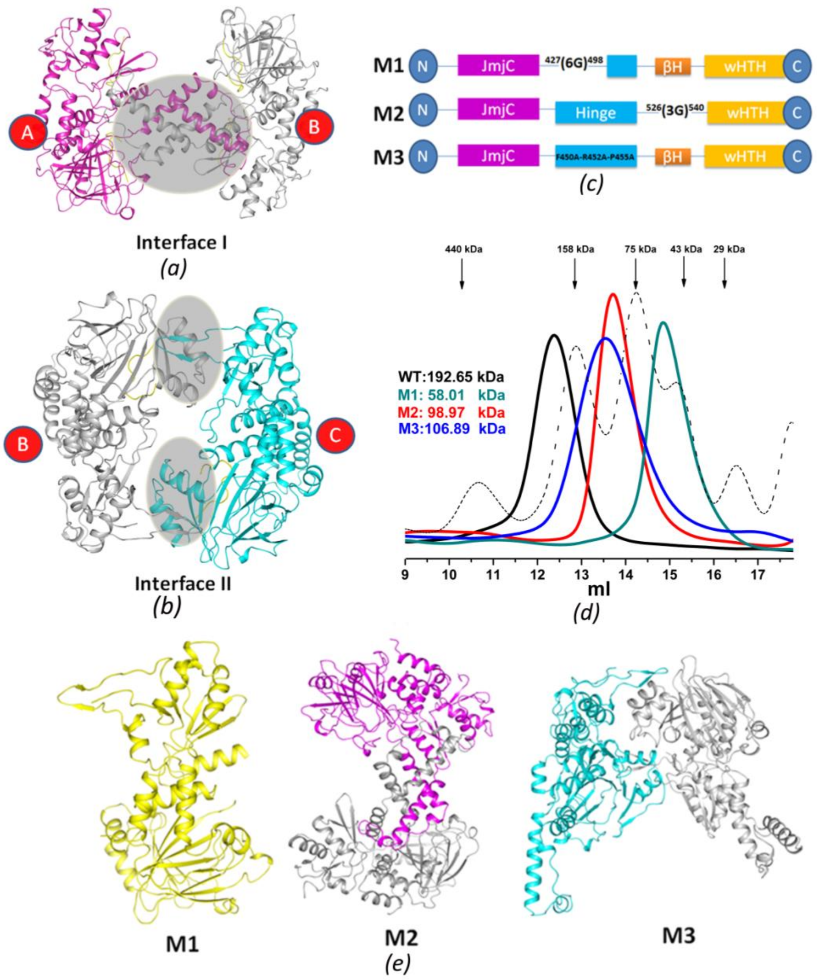

Figure S2 Structural models of mutant proteins of NO66. (*a*) Interface I (left panel) and (*b*) II (right panel) of NO66 are shown. (*c*) Schematic drawing of the mutants used to study NO66 substrate binding and enzymatic activity. (*d*) Size exclusion chromatography analyses of wild type and mutants of NO66. The molecular weights of WT, mutants M1 (residues 427-498 was replaced by six glycine residues), M2, (residues 526-540 was replaced by three glycine residues) and M3 (F450,R452 and P455 was substituted with alanine) are caculated from the elution volume based on the standard curve. WT, M1, M2 and M3 are eluted as the monomer (cyan), dimer (red), and dimer (blue), respectively. The chromatographic separation of the standard proteins is shown in black dash line and the theoretical molecular weights are shown above. (*e*) Structural models of mutant proteins of NO66. M1 is generated by deletion of interface I of NO66 and exists as a monomer. M2 is generated by deletion of interface II and exists as a dimer linked by interface I. M3 is generated by mutating three amino acid residues in interface I, and exists as a dimer connected by interface II.

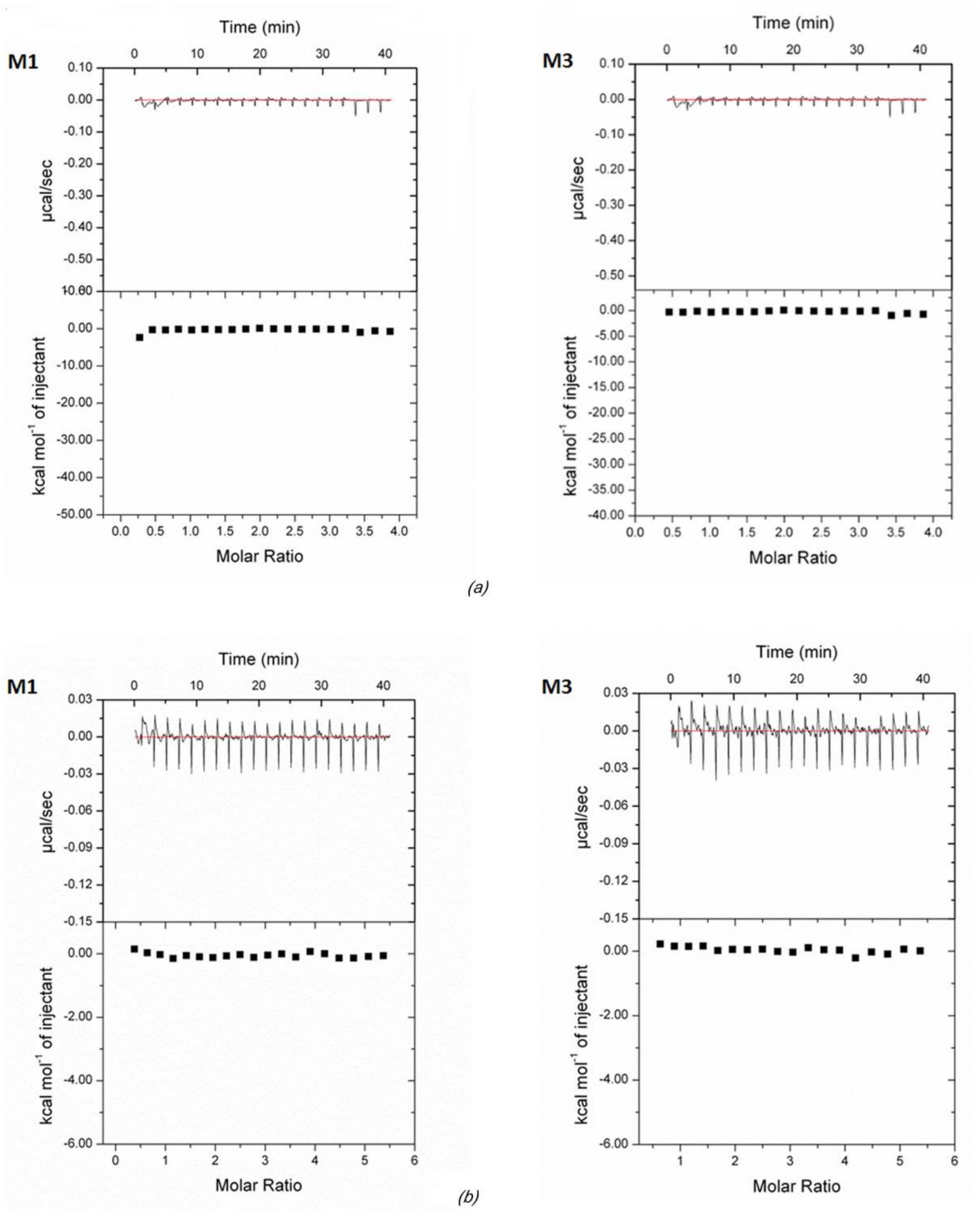

Figure S3 Binding of mutant proteins of NO66 to GST-Rpl8$^{193\text{-C}}$ and αKG. (*a*) Binding of M1 and M3 to GST-Rpl8$^{193\text{-C}}$. The ITC method was used to analyze the binding affinity. (*b*) Binding of M1 and M3 to αKG. The ITC method was used to analyze the binding affinity.

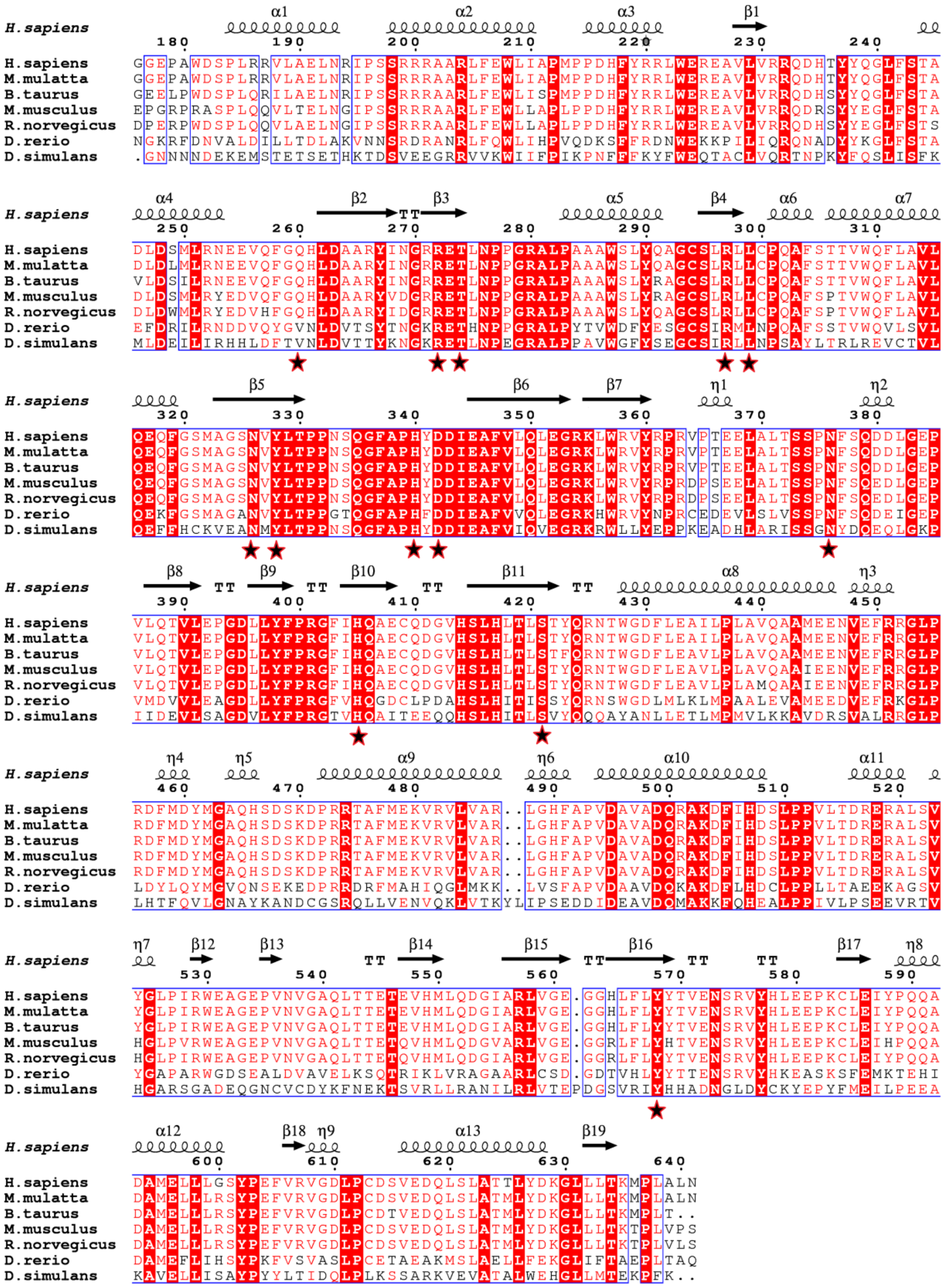

Figure S4 Structure-based sequence alignment of NO66 from different species. The secondary structure elements are labeled according to the structure of NO66$^{176\text{-}C}$. The residues involved in the substrate binding are highlighted with a pentagram. The accession numbers of protein sequences are: *Homo sapiens* (NP_078920, human), *Macaca mulatta*

(XP_001090820, rhesus), *Bos taurus* (NP_001093172, cattle), *Mus musculus* (NP_076122, mouse), *Rattus norvegicus* (NP_001101510, rat), *Danio rerio* (NP_001082857, fish), and *Drosophila simulans* (XP_002106114, fly).

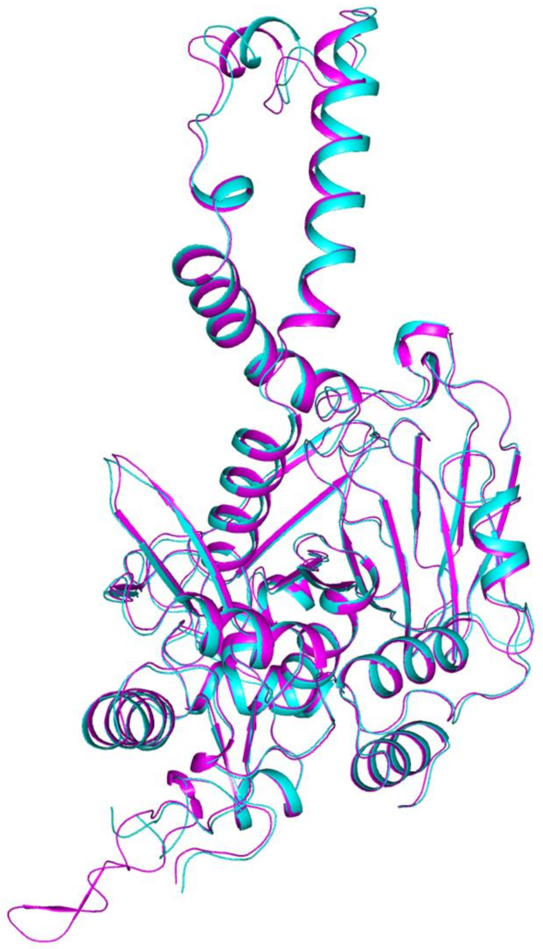

Figure S5 Superimposition of monomeric NO66 in apo-form (PDB ID: 4E4H, magenta) to M2 (cyan).

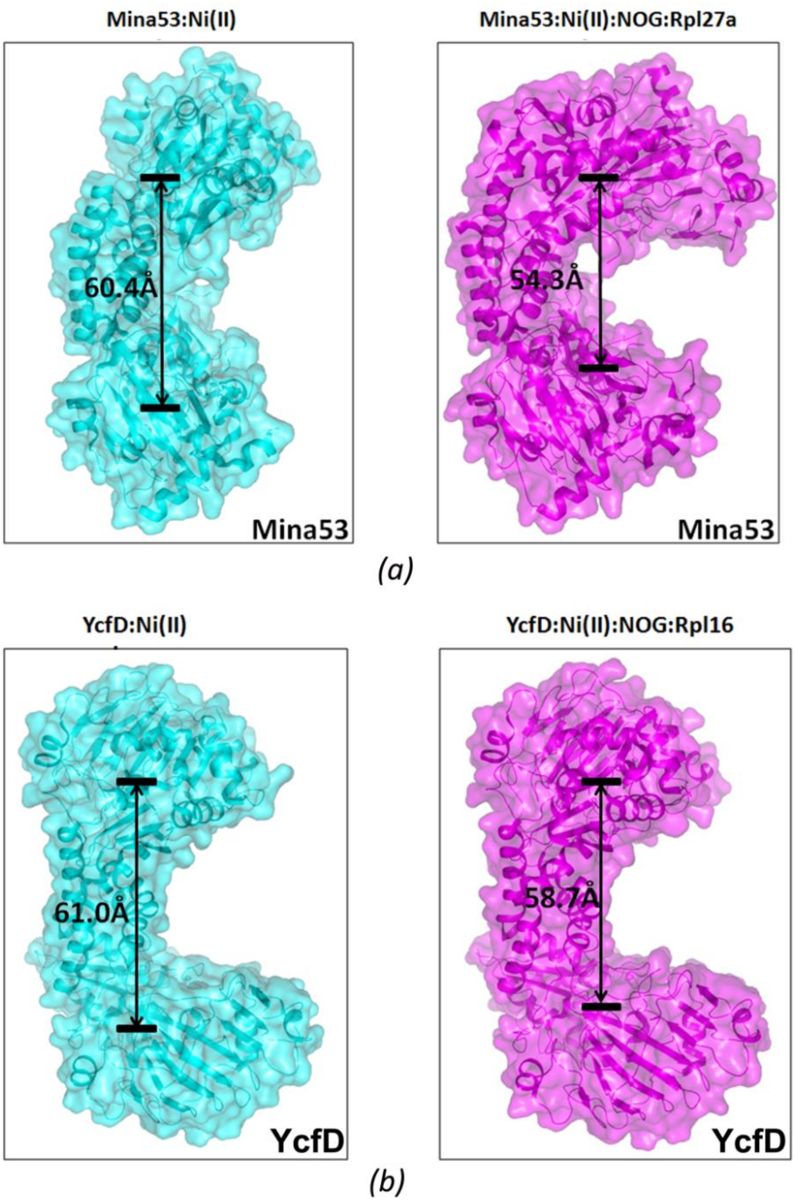

Figure S6 Conformational changes of Mina53 and YcfD induced by substrate binding. (*a*) Surface representation of dimeric Mina53 in apo-form (PDB ID: 4BU2, left panel) and in substrate binding form (PDB ID: 4BXF, right panel). (*b*) Surface representation of dimeric YcfD in apo-form (PDB ID: 4CSW, left panel) and in substrate binding form (PDB ID: 4CUG, right panel). The distances between the active sites in the dimeric Mina53 and YcfD in apo-form or in substrate binding form are indicated, respectively.

Table S1 Comparison of the average B-factor values between NO66[176-C]-Rpl8[204-224] complex structure in nateve state (4Y3O) and cross-lingking structures reported previously (4CCM, 4CCN, 4CCO).

PDB ID		4Y3O (2.2Å)	4CCM (2.51Å)	4CCN (2.23Å)	4CCO (2.3Å)
B-factor	Whole chain	24.47	54.44	37.35	36.9
	Rpl8 peptides	37.65	89.35	43.85	63.7
	"NHQH" residues	27.76	86.3	36.3	58.4

Table S2 List of proteins containing sequences similar to the consensus motif recognized by NO66.

The matched similar motif residues are highlighted in red.

Protein name	Match motif sequence
RNA-binding protein 12B	SSEKMQARSQSRERGDHSHLFDS KDPPIY
GC-rich promoter binding protein 1-like 1	RGHDGMSQRSGGGTGNHRHW NGSFHSRK
Cbp/p300-interacting transactivator, with Glu/Asp-rich carboxy-terminal domain, 2	HAFNALMGEHIHYGAGNMNAT SGIR
TATA-binding protein-associated factor 172	CILAGDHCHRAQEYA RSKLA
Melanoma antigen preferentially-expressed in tumors	PFTCLPLGVLMKGQHLHLET
Ubiquitin carboxyl-terminal hydrolase 40	ELKLMKELGNHLHFLFGS
Tartrate-resistant acid phosphatase type 5	KVPWYVLAGNHDHLGNVSAQ
Membrane-bound transcription factor site-1 protease precursor	EGNHLHRYSKVLEAHLGDPK
BTAF1 RNA polymerase II, B-TFIID transcription factor-associated	SICILAGDHCHRAQEYARSK
Methenyltetrahydrofolate synthase domain-containing protein	KDVTLQGEHQHLPEPGCQQT
Spermine oxidase	RGPEIEPRGEGDHNHDTGEG
Putative uncharacterized protein DKFZp762N1113	GKLVPAGDHWHLSDLGQLQS
WEE1 homolog	QNALREVYAHAVLGQHSHVV
TOP1 protein	MSGDHLHNDSQIEADFRLNDSH KHKDKHKD
SPOC domain-containing protein 1	HGFGRGQHFHRDSCPHQALL

Centrosomal protein of 63 kDa	EFKNTEFKPTHGQHRHDGIKTEHYKTDLHS
WW domain-containing adapter protein with coiled-coil	SSKSHPSSGDHRHEKMRDAG
Zinc finger protein 446	SPPLAAQSPEGNHGHQEPAS
TP53-target gene 1 protein	EPLFAAPGEHLHQCFVKESY
RNA polymerase-associated protein LEO1	KAPLQGDHNHLFIRQGTGLQ
